# Epididymal abnormalities associated with sperm obstruction

**DOI:** 10.1590/S1677-5538.IBJU.2022.9907.1

**Published:** 2022-03-01

**Authors:** Rodrigo R. Vieiralves

**Affiliations:** 1 Hospital Federal da Lagoa Rio de Janeiro RJ Brasil Serviço de Urologia, Hospital Federal da Lagoa, Rio de Janeiro, RJ, Brasil

## COMMENT

The testicular descent process is a complex event, occurring defectively in 1% to 4% of full- term newborn boys and 1% to 45% of preterm newborns (1). Cryptorchia is a common genital anomaly not fully understood. Much has been studied on the histological development of cryptorchid testes with many previous publications. More than 40 years ago, germ cell counts, and the analysis of spermatogonial development associated the cryptorchidism to male fertile potential, with strong data showing that abnormal germ cell development is often present (2). In addition, anomalies of the epididymis, vaginal process and gubernaculum have been reported with a potential impact on fertility, especially regarding the anatomical relationship between the epididymis and the testis. This may be abnormal in boys with cryptorchidism (3), but the reported frequency ranges widely from 16% to 75% probably because this anomaly is difficult to be characterized and classified (4, 5). In this context, which criteria, then, related to the patterns of epididymal anomalies, would be associated with a possible obstruction, thus impacting fertility?

In this interesting study, with original figures and clear tables, the authors evaluated a number of epididymal abnormalities associated with sperm obstruction in patients with undescended testis according to: testicular position, patient age and patency of the vaginal process (6). For this, in a period of nine years (2011-2020), 87 patients with cryptorchidism who underwent orchidopexy had their condition retrospectively analyzed. For the relationship between the testis and the epididymis, the authors used a previous classification (7, 8): Type I - epididymis adhered to the testis in the head and tail region; Type II - epididymis fully adhered to the testis; Type III - epididymis inserted in the testicle only in the head; Type IV - epididymis adhered to the testis only in the tail; Type V - no visible connection between testis and epididymis; and Type VI - epididymal atresia. Type I and II relationships were considered normal, while the other types are considered epididymal anomalies. Those with head disjunction and total epididymal disjunction (Type IV and V) were locked together, as, in theory, it can lead to infertility due to the presence of a sperm obstruction preventing the passage of sperm to the epididymis, if present bilaterally.

Based on this classification, the authors identified epididymal anomalies associated with sperm obstruction in almost 20% of cases, occurrence that is unrelated with age, testicular position or permeability of the vaginal process. That said, it is clear a potential impact of this pathology on the male reproductive function, which may be associated with obstructive azoospermia. Despite this initial conclusion, an impossibility of histological analysis (observing the patency of the epididymal ducts - obviously not performed for ethical reasons) and the lack of long-term follow-up to verify if they developed infertility, are limitations brought up in the study that actually compromise more solid conclusions.

Despite the great enthusiasm that studies like these bring for understanding a complex process of anomalies of the epididymal testicular junction, condition in theory possibly associated with with obstructive azoozpermia, a long journey ahead awaits us. The authors have a recognized work in this field and surely, future studies confirming epididymal obstruction and long term clinical follow-up will help us to reach clear answers.
